# Epigenomic profiling of prostate cancer identifies differentially methylated genes in *TMPRSS2*:*ERG* fusion-positive versus fusion-negative tumors

**DOI:** 10.1186/s13148-015-0161-6

**Published:** 2015-12-12

**Authors:** Milan S. Geybels, Joshi J. Alumkal, Manuel Luedeke, Antje Rinckleb, Shanshan Zhao, Irene M. Shui, Marina Bibikova, Brandy Klotzle, Piet A. van den Brandt, Elaine A. Ostrander, Jian-Bing Fan, Ziding Feng, Christiane Maier, Janet L. Stanford

**Affiliations:** Division of Public Health Sciences, Fred Hutchinson Cancer Research Center, Seattle, WA USA; Department of Epidemiology, GROW School for Oncology and Developmental Biology, Maastricht University, Maastricht, The Netherlands; Division of Hematology and Medical Oncology, Knight Cancer Institute, Oregon Health and Science University, Portland, OR USA; Institute of Human Genetics and Department of Urology, Faculty of Medicine, University of Ulm, Ulm, Germany; Biostatistics and Computational Biology Branch, National Institute of Environmental Health Sciences, NC Research Triangle Park, USA; Illumina, Inc., San Diego, CA USA; Cancer Genetics Branch, National Human Genome Research Institute, NIH, Bethesda, MD USA; MD Anderson Cancer Center, Houston, TX USA; Department of Epidemiology, School of Public Health, University of Washington, Seattle, WA USA; Present Address: AnchorDx Corp., Guangzhou, 510300 People’s Republic of China

**Keywords:** DNA methylation, CpG site, Epigenetics, Epigenomic profiling, Prostate cancer, Gene fusion, *TMPRSS2*, *ERG*, Tumor tissue, Unsupervised clustering, mRNA expression, *C3orf14*, *CACNA1D*, *GREM1*, *KLK10*, *NT5C*, *PDE4D*, *RAB40C*, *SEPT9*, *TRIB2*, TCGA

## Abstract

**Background:**

About half of all prostate cancers harbor the *TMPRSS2*:*ERG* (*T2E*) gene fusion. While *T2E*-positive and *T2E*-negative tumors represent specific molecular subtypes of prostate cancer (PCa), previous studies have not yet comprehensively investigated how these tumor subtypes differ at the epigenetic level. We therefore investigated epigenome-wide DNA methylation profiles of PCa stratified by *T2E* status.

**Results:**

The study included 496 patients with clinically localized PCa who had a radical prostatectomy as primary treatment for PCa. Fluorescence in situ hybridization (FISH) “break-apart” assays were used to determine tumor *T2E*-fusion status, which showed that 266 patients (53.6 %) had *T2E*-positive PCa. The study showed global DNA methylation differences between tumor subtypes. A large number of differentially methylated CpG sites were identified (false-discovery rate [FDR] Q-value <0.00001; *n* = 27,876) and DNA methylation profiles accurately distinguished between tumor *T2E* subgroups. A number of top-ranked differentially methylated CpGs in genes (FDR Q-values ≤1.53E−29) were identified: *C3orf14*, *CACNA1D*, *GREM1*, *KLK10*, *NT5C*, *PDE4D*, *RAB40C*, *SEPT9*, and *TRIB2*, several of which had a corresponding alteration in mRNA expression. These genes may have various roles in the pathogenesis of PCa, and the calcium-channel gene *CACNA1D* is a known *ERG*-target. Analysis of The Cancer Genome Atlas (TCGA) data provided confirmatory evidence for our findings.

**Conclusions:**

This study identified substantial differences in DNA methylation profiles of *T2E*-positive and *T2E*-negative tumors, thereby providing further evidence that different underlying oncogenic pathways characterize these molecular subtypes.

**Electronic supplementary material:**

The online version of this article (doi:10.1186/s13148-015-0161-6) contains supplementary material, which is available to authorized users.

## Background

In 2005, Tomlins et al. identified the fusion of two genes, *ERG* and *TMPRSS2*, as a common somatic alteration in prostate cancer (PCa) [1]. Formation of the *TMPRSS2*:*ERG* (*T2E*) gene fusion results in overexpression of *ERG*, a known oncogene and member of the *ETS* transcription factor family [1]. *TMPRSS2* is an androgen-regulated gene that encodes a serine protease and is preferentially expressed in the prostate [2]. The gene fusion can result from a chromosomal translocation or interstitial deletion [3]. About 50 % of PCa patients of European ancestry harbor *T2E*-positive tumors, but lower frequencies have been reported in men of African or Asian ancestry [4]. The *T2E* gene fusion is an early event in PCa, and fusion-positive tumors are believed to represent a distinct molecular subtype of PCa involving activation of specific oncogenic pathways [2, 3, 5–13].

The gene fusion may have clinical implications. It has been shown that the *T2E* transcript can be detected in urine and that this represents a specific biomarker for the detection of PCa [14]. Several studies have also investigated fusion status in relation to PCa outcomes, but a recent meta-analysis of 48 studies showed no evidence of an association with recurrence-free or disease-specific survival [15]. Although the clinical relevance of molecular subtyping of PCa by *T2E* status is unknown, it might allow patient stratification for different management strategies [16, 17].

DNA methylation of cytosines in CpG dinucleotides is an epigenetic mechanism for control of gene transcription [18, 19]. CpG sites are commonly found in clusters called CpG islands, which are often in gene promoter regions. While CpGs outside islands are usually methylated, CpGs in islands in gene promoter regions are typically unmethylated [18]. Hypermethylation of gene promoter regions can lead to transcriptional silencing, but DNA methylation changes outside gene promoter regions (e.g., the gene body) can also play critical roles in the regulation of gene activity and genomic stability [20, 21]. Both losses and gains of DNA methylation have been associated with cancer, including PCa [18, 22].

There is preliminary evidence from two small studies that *T2E* status is associated with changes in DNA methylation [23, 24]. Both studies used an epigenome-wide approach that focused on larger differentially methylated regions (≥500 bp). Using this approach, many key (de)methylated CpG sites that are critical for regulating gene expression may have been missed [25]. Further research is therefore needed to precisely assess DNA methylation at single CpG resolution in fusion-positive versus fusion-negative prostate tumors. Another limitation of these previous analyses is the small sample size. The total number of fusion-positive and fusion-negative tumors in the first and second study was 9 and 37, respectively.

The present study investigated epigenome-wide DNA methylation profiles in *T2E*-fusion-positive versus *T2E*-fusion-negative prostate tumors in a large population-based cohort of 496 patients to identify differentially methylated CpG sites. We integrated methylation results with gene expression data, from the same patients’ tumor samples, to investigate the potential effects of differential DNA methylation on mRNA expression levels. Further, data from The Cancer Genome Atlas (TCGA) were used to independently validate our methylation findings.

## Results

The study included 496 PCa patients who received radical prostatectomy as primary treatment for clinically localized disease. Of these, 266 (53.6 %) were *T2E*-fusion-positive (Table 1). Fusion-positive PCa was associated with younger ages at cancer diagnosis, European-American race, and lower Gleason scores.

**Table 1:** Characteristics of PCa patients by *T2E*-fusion status

	*T2E*-negative PCa (*n* = 230)			*T2E*-positive PCa (*n* = 266)			
Variables	No.	%	Mean (SD)	No.	%	Mean (SD)	*P* value^a^
Age at diagnosis (years)	–	–	59.5 (7.1)	–	–	56.8 (6.8)	<0.01
Race							<0.01
African-American	29	12.6	–	14	5.3	–	–
European-American	201	87.4	–	252	94.7	–	–
Gleason score							<0.01
≤6	92	40.0	–	141	53.0	–	–
7 (3 + 4)	89	38.7	–	93	35.0	–	–
7 (4 + 3)	29	12.6	–	15	5.6	–	–
≥8	20	8.7	–	17	6.4	–	–
Pathological stage							0.70
Local (pT1/pT2)	159	69.1	–	179	67.3	–	–
Regional (pT3)	71	30.9	–	87	32.7	–	–
PSA (ng/mL) at diagnosis							0.06
<4	26	12.0	–	52	20.6	–	–
4– < 10	138	63.6	–	156	61.9	–	–
10– < 20	38	17.5	–	28	11.1	–	–
≥20	15	6.9	–	16	6.3	–	–

*T2E TMPRSS2*:*ERG*, *PCa* prostate cancer, *SD* standard deviation, *PSA* prostate-specific antigen

^a^A *t* test (age at diagnosis) or chi-square test (all categorical variables) was used

**Fig. 1:**
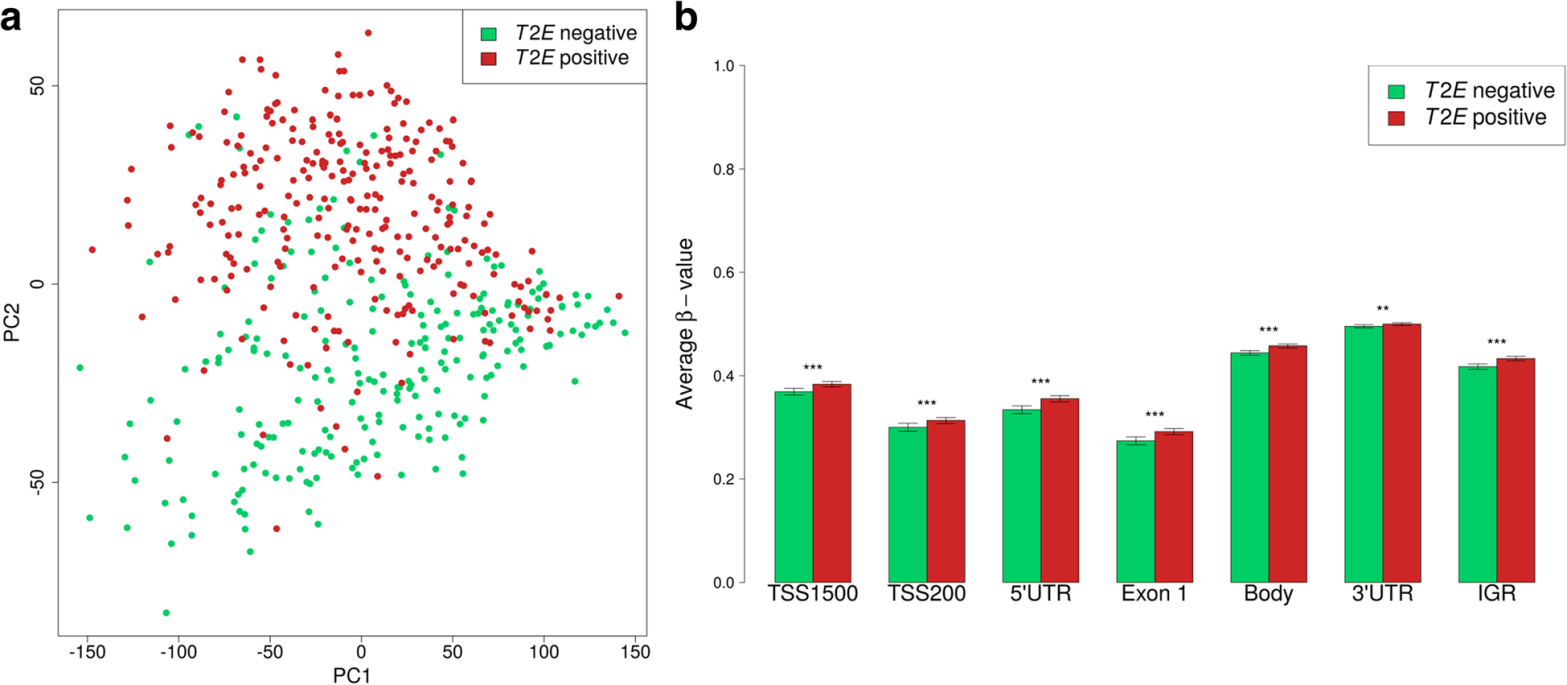
DNA methylation and *T2E* status. **a** Principal component (PC) analysis plot based on the 5000 most variable CpG sites in the dataset. **b** Average DNA methylation level of the same 5000 CpG sites, by genetic location (Illumina annotation). Statistically significant differences are highlighted; *P value <0.05, **<0.01, or ***<0.001

A principal component analysis of DNA methylation levels was conducted. The 5000 most variable CpG sites in the dataset were used as input for this analysis. In a plot of principal component 1 vs. 2, *T2E*-positive and *T2E*-negative tumors were separated, suggesting that these tumor subtypes have a distinct DNA methylome (Fig. 1a). After that, we calculated the average DNA methylation level of the 5000 most variable CpG sites in *T2E*-positive and *T2E*-negative PCa, stratified by genetic location. This showed that DNA methylation levels were higher in *T2E*-positive tumors (*P* value <0.05; Fig. 1b).

**Fig. 2:**
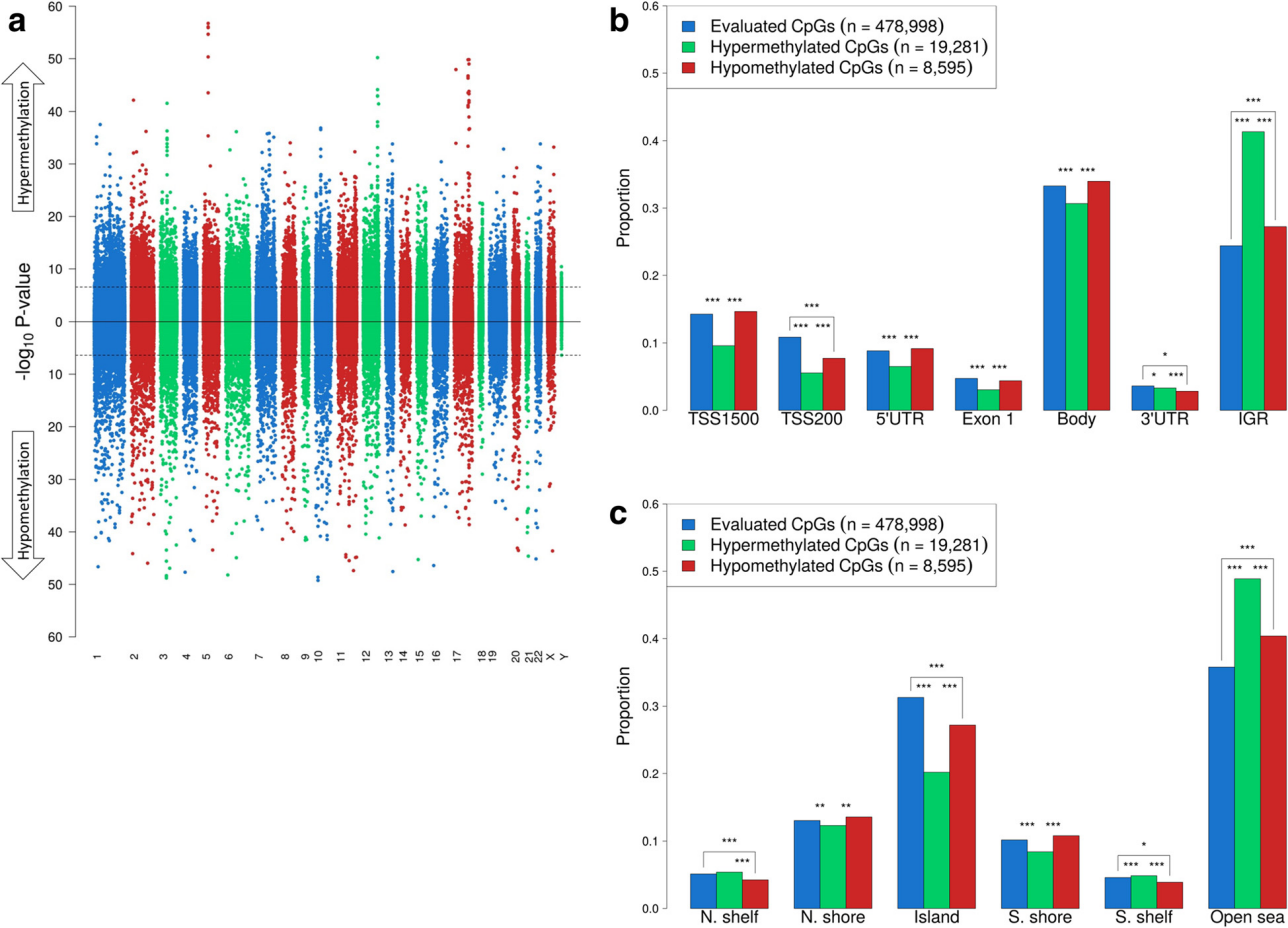
Differentially methylated CpG sites in fusion-positive versus fusion-negative PCa. **a** Manhattan plot of DNA methylation. The horizontal axis shows the chromosomes. A 10,000-bp “gap” was added between each chromosome to aid visualization. The *dashed line* represents the *P* value that corresponds to the FDR Q-value threshold for statistical significance of 0.00001. In total, 19,281 hypermethylated and 8595 hypomethylated CpGs reached statistical significance. The frequency of all evaluated and hyper- and hypomethylated CpG sites by **b** gene region and **c** epigenetic region. Genetic and epigenetic locations are based on Illumina annotation. Statistically significant differences are highlighted; *P value <0.05, **<0.01, or ***<0.001

Figure 2a shows a Manhattan plot, which highlights the distribution of differentially methylated CpG sites across the genome. There were 27,946 differentially methylated CpGs (false-discovery rate [FDR] Q-value <0.00001), including 19,281 CpGs (69 %) that were hypermethylated and 8595 CpGs (31 %) that were hypomethylated in fusion-positive versus fusion-negative PCa. Figure 2b shows the frequency of all evaluated and the significantly hyper- and hypomethylated CpG sites by genetic location. Similarly, Fig. 2c shows these frequencies by epigenetic location. These figures illustrate that the frequencies of hyper- and hypomethylated CpGs in many gene and epigenetic locations differ from the frequencies of all evaluated CpGs in these locations. In particular, hypermethylated CpGs were enriched in intergenic and open sea regions but underrepresented in CpG islands and promoter regions.

**Fig. 3:**
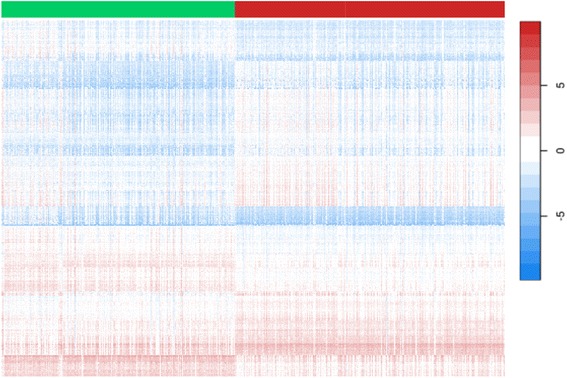
Heat map of DNA methylation M-values in fusion-positive versus fusion-negative PCa, based on supervised clustering. The *columns* represent the prostate tumor samples (fusion-positive is shown under the *red* bar and fusion-negative is shown under the *green bar*). The heat map includes 3101 differentially methylated CpG sites (*T2E* ^+^ vs. *T2E* ^─^) with FDR Q-value <0.00001 and a mean methylation difference of at least 10 % between tumor types (*rows*). Higher methylation levels are shown in *red* and lower methylation levels are shown in *blue* (*white* is intermediate)

Of the 27K significant CpG sites, 3103 had a mean methylation difference of at least 10 % between *T2E* subtypes (Additional file 1). Figure 3 shows a heat map of these 3K CpGs based on supervised clustering. This again shows that fusion-positive and fusion-negative prostate tumors have distinct epigenetic profiles. These differentially methylated CpG sites involved 1962 genes. This set of genes was used for gene ontology (GO) analysis. We found that seven of the top ten identified GO-associated biological pathways were related to developmental processes (not shown).

**Fig. 4:**
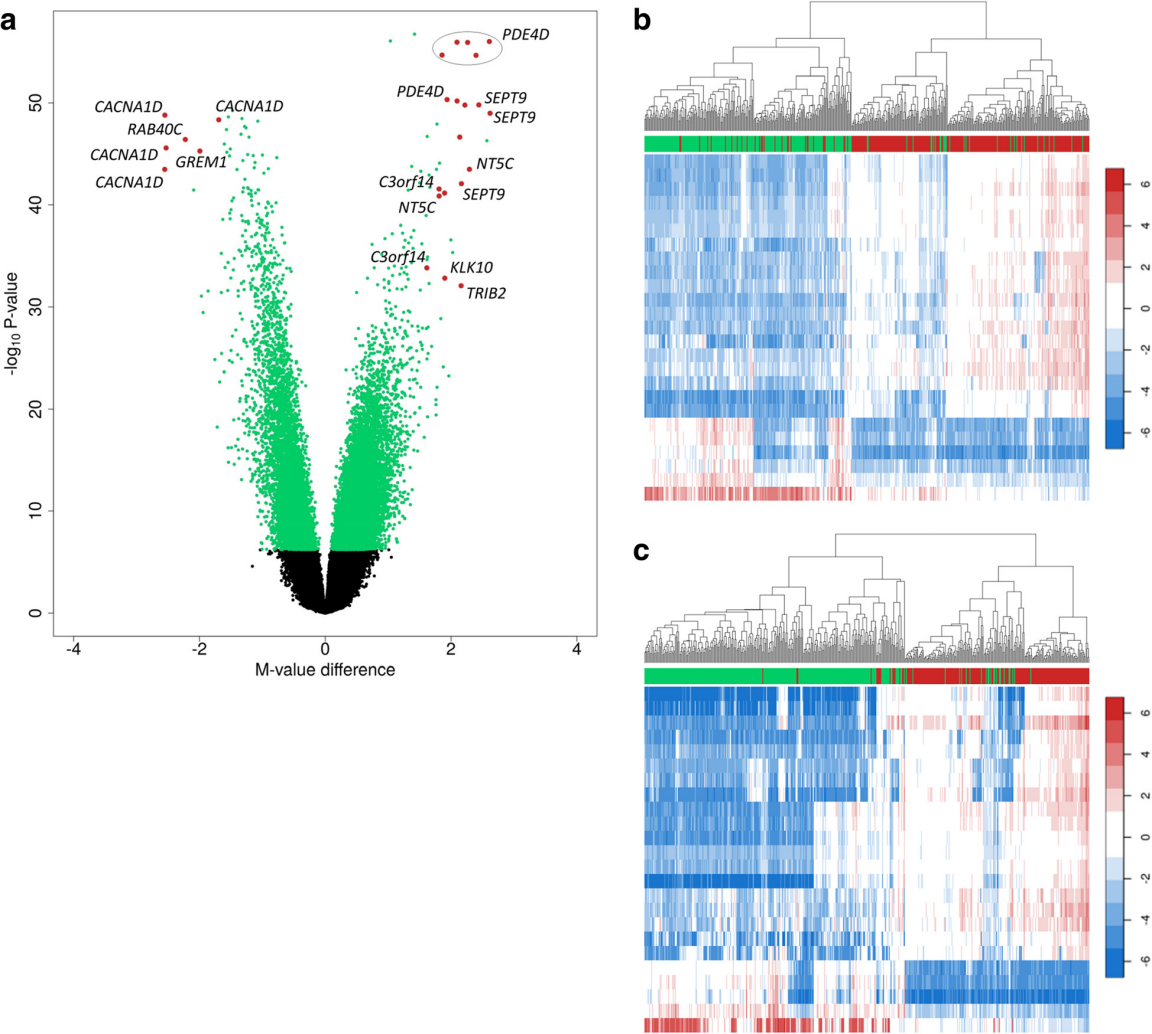
Twenty-five top-ranked differentially methylated CpG sites in fusion-positive versus fusion-negative PCa. **a** Volcano plot of DNA methylation. Differentially methylated CpGs (FDR Q-value <0.00001; *n* = 27,946) are displayed in *green* or *red*. The 25 red-labeled CpGs had a mean methylation difference of at least 25 % between tumor types, and the figure shows the genes these CpG sites map to. Four of the 25 CpGs were intergenic. **b** Unsupervised clustering using the 25 top differentially methylated CpG sites (*rows*) with FDR Q-value <0.00001 and a mean methylation difference of at least 25 % between prostate tumor types, in our cohort. The *columns* represent the prostate tumor samples (fusion-positive is shown under the *red* bar and fusion-negative is shown under the *green bar*). Higher methylation levels are displayed in *red* and lower methylation levels are shown in *blue* (*white* is intermediate). Two main clusters were identified, one that consisted primarily of fusion-positive tumors (89 %) and the other that consisted mostly of fusion-negative tumors (87 %). **c** Unsupervised clustering using the top CpG sites in TCGA (same approach as in **b**). One of the CpG sites (*GREM1* cg17312492) was not represented in TCGA data, and the analysis therefore only included 24 CpG sites. Similar to our results, clustering using these CpG sites clearly separated fusion-positive from fusion-negative PCa. One of the two clusters contained 89 % of fusion-positive tumors and the other cluster contained 95 % of fusion-negative tumors

Next, we focused on the differentially methylated CpGs with the largest mean methylation difference between fusion-negative and fusion-positive PCa (≥25 %). Twenty-five such top-ranked CpGs were identified (Fig. 4a, Table 2), of which 19 were hypermethylated and six were hypomethylated in fusion-positive versus fusion-negative PCa. Fifteen of the hypermethylated CpGs were in six genes: *PDE4D* (*n* = 6), *SEPT9* (*n* = 3), *NT5C* (*n* = 2), *C3orf14* (*n* = 2), *KLK10* (*n* = 1), and *TRIB2* (*n* = 1); all six hypomethylated CpGs were in three genes: *CACNA1D* (*n* = 4), *RAB40C* (*n* = 1), and *GREM1* (*n* = 1). Four hypermethylated CpGs were intergenic including one CpG on chromosome 12 and three CpGs on chromosome 17. Three of the 25 CpGs were in single nucleotide polymorphism (SNP) loci: *PDE4D* cg22706610, cg13468945, and *GREM1* cg17312492, and the associations of these specific CpGs therefore need to be interpreted with caution.

**Table 2:** Top-ranked differentially methylated CpGs in *T2E*-fusion-positive versus *T2E*-fusion-negative prostate tumors

CpG ID		Chromosome	Gene	Location	CpG island	Mean β *T2E*-negative	Mean β *T2E*-positive	Mean β difference	Q-value
Hypermethylation									
cg11258089		5	*PDE4D*	Body; TSS200	Yes	0.20	0.53	0.33	1.19E−51
cg22706610		5	*PDE4D*	Body; TSS200	Yes	0.22	0.50	0.28	1.19E−51
cg26870744		5	*PDE4D*	Body; TSS200	Yes	0.17	0.43	0.27	1.19E−51
cg07190535		5	*PDE4D*	Body; TSS1500	Yes	0.26	0.53	0.27	1.57E−50
cg08554295		5	*PDE4D*	Body; TSS1500	No	0.18	0.46	0.29	1.57E−50
cg16261871		5	*PDE4D*	Body; TSS200	Yes	0.25	0.52	0.28	2.83E−46
cg19694010		12	–	IGR	Yes	0.19	0.45	0.27	3.45E−46
cg15044248		17	*SEPT9*	TSS200; 5′ UTR; body	Yes	0.11	0.38	0.26	7.05E−46
cg14087806		17	–	IGR	Yes	0.19	0.49	0.30	7.05E−46
cg06848185		17	*SEPT9*	TSS1500; 5′ UTR; body	Yes	0.10	0.36	0.26	3.84E−45
cg13468945		17	–	IGR	Yes	0.19	0.47	0.29	4.20E−43
cg08378505		17	*NT5C*	Body	Yes	0.15	0.45	0.30	3.41E−40
cg17300544		17	*SEPT9*	TSS200; 5′ UTR; body	Yes	0.18	0.47	0.29	7.49E−39
cg26896572		3	*C3orf14*	TSS1500	Yes	0.26	0.53	0.27	2.28E−38
cg21454031		17	–	IGR	No	0.22	0.48	0.26	5.03E−38
cg10520594		17	*NT5C*	Body	Yes	0.19	0.44	0.25	9.43E−38
cg15059608		3	*C3orf14*	TSS1500	No	0.32	0.58	0.25	3.36E−31
cg21869055		19	*KLK10*	5′ UTR	Yes	0.26	0.51	0.25	3.06E−30
cg25623934		2	*TRIB2*	Exon 1; body	Yes	0.22	0.49	0.27	1.53E−29
Hypomethylation									
cg08480458		3	*CACNA1D*	Body	Yes	0.47	0.15	0.32	5.42E−45
cg15473186		3	*CACNA1D*	Body	Yes	0.57	0.31	0.27	1.28E−44
cg06717750		16	*RAB40C*	Body	No	0.80	0.52	0.28	6.87E−43
cg12668309		3	*CACNA1D*	Body	Yes	0.33	0.08	0.26	4.18E−42
cg17312492		15	*GREM1*	5′ UTR	Yes	0.43	0.17	0.26	7.96E−42
cg14337339		3	*CACNA1D*	Body	Yes	0.43	0.12	0.31	3.41E−40

The table shows differentially methylated CpGs (FDR Q-value <0.00001) that have a mean methylation (β-value) difference of at least 25 % between fusion-positive versus fusion-negative PCa

*T2E TMPRSS2*:*ERG*, *TSS1500* 200 to 1500 base pairs upstream of the transcription start site, *TSS200* 200 base pairs upstream of the transcription start site, *UTR* untranslated region, *IGR* intergenic region

DNA methylation at adjacent CpG sites is typically correlated. Correlations between the methylation levels of the CpG sites in Table 2 that were in the same gene (i.e., *PDE4D*, *SEPT9*, *NT5C*, *C3orf14*, and *CACNA1D*) were ≥0.9. The three intergenic CpGs on chromosome 17 (Table 2) were in the same 803-bp region, and methylation levels of these CpGs were also highly correlated (*r*^2^ ≥ 1.0). Further, all 25 top CpGs were in larger differentially methylated regions that included multiple additional CpG sites for which DNA methylation levels were correlated (*r*^2^ ≥ 0.8; median number of CpGs per region = 5, range 2–10).

A hierarchical clustering analysis based on the methylation levels of the 25 top-ranked CpGs identified two main clusters, one that consisted primarily of fusion-positive tumors (89 %) and the other that consisted mostly of fusion-negative tumors (87 %; Fig. 4b). These data suggest that epigenetic profiles based on these 25 CpG sites can separate fusion-positive from fusion-negative prostate tumors.

Next, we investigated the associations between *T2E* status and methylation of the 25 top CpGs in subgroups of European-American (*n* = 453) and African-American patients (*n* = 43). Although the analysis of African-American men was underpowered, all associations were in the same direction in both subgroups, suggesting that these associations are not substantially different for these two ancestry groups. In addition, associations between fusion status and DNA methylation of the 25 top-ranked CpGs were investigated in subgroups based on Gleason score (≤7 [3 + 4] vs. ≥7 [4 + 3]) and age at diagnosis (<60 vs. ≥60 years), which showed no substantial differences.

mRNA expression levels of the nine genes containing the top-ranked differentially methylated CpGs (Table 2) were investigated using the same patients’ tumor tissue samples. Methylation levels of CpG sites in six of these genes were correlated with gene expression levels (*P* value <0.05): *C3orf14* (range *r*^2^ −0.60, −0.63), *CACNA1D* (range *r*^2^ −0.39, −0.45), *GREM1* (*r*^2^ −0.32), *NT5C* (*r*^2^ −0.42), *SEPT9* (range *r*^2^ 0.24, 0.31), and *TRIB2* (*r*^2^ −0.24). In addition, the expression of *ERG* was investigated and we confirmed its overexpression in fusion-positive compared to fusion-negative PCa (log_2_ fold change = 1.92, *P* value = 1.82E−68).

In a final analysis, we aimed to confirm our methylation findings in The Cancer Genome Atlas (TCGA) dataset. Because *T2E*-fusion status was not directly determined in TCGA using fluorescence in situ hybridization (FISH), we used *ERG* mRNA overexpression as a proxy for positive fusion status, as described previously [26–28]. As such, we found that 187 of the 468 TCGA prostate tumor samples available for analysis were *T2E*-fusion-positive (40 %). First, we focused on all 27K significant CpG sites in our study. This showed that the majority of hyper- (91 %) and hypomethylated (98 %) CpGs in our study, with available methylation data in TCGA, were similarly associated with fusion status in TCGA (*P* value <0.05). Second, the 25 top-ranked CpGs in our study were examined in more detail. Methylation data were available for 24 of the 25 CpGs; *GREM1* cg17312492 was not available in the TCGA dataset. The 24 CpGs were similarly differentially methylated by *T2E* status in TCGA (*P* values ≤5.44E−37); mean methylation differences between patient groups for these CpGs ranged from 24 to 49 % (mean = 36 %). Similarly as in our discovery cohort, hierarchical clustering using the methylation levels of these CpGs identified two main clusters, one that consisted primarily of fusion-positive tumors (89 %) and the other that consisted mostly of fusion-negative tumors (95 %; Fig. 4c). Our demonstration of these results across two cohorts suggests that these top differentially methylated CpGs and genes are strongly and robustly associated with *T2E* status.

## Discussion

The present study identified substantial DNA methylation differences in *T2E*-positive and *T2E*-negative prostate tumors. We found global DNA methylation differences and identified a large number of differentially methylated CpG sites. Fusion-positive and fusion-negative prostate tumors could be accurately distinguished by their DNA methylation profiles. Several of the top-ranked genes identified in this study showed aberrant DNA methylation levels that correlated with altered mRNA expression levels, suggesting a role for DNA methylation in regulating the transcription of these genes. Analysis of TCGA data provided confirmatory evidence for our findings.

A number of previous studies found that *CACNA1D* expression correlates with *ERG* overexpression in *T2E*-fusion-positive PCa, suggesting that *CACNA1D* is an *ERG* target gene [5, 7–13]. *CACNA1D* is a calcium-channel gene that encodes the l-type calcium-channel alpha 1D subunit (Ca_v_1.3), which is involved in several biological processes including cell signaling and calcium homeostasis [29, 30]. This epigenome-wide analysis of fusion-positive versus fusion-negative PCa identified *CACNA1D* as one of nine top-ranked differentially methylated genes and confirmed that the gene transcript is overexpressed in fusion-positive PCa. A CpG island in the gene body of this gene had lower methylation levels in fusion-positive than fusion-negative PCa. Interestingly, a previous study of DNA methylation in fusion-positive (*n* = 17) versus fusion-negative PCa (*n* = 20) found two larger hypomethylated regions, 500 bp in size, that were in the same genomic region as the hypomethylated CpG sites in the gene body of *CACNA1D* identified in the present study [23].

While promoter hypermethylation is often associated with transcriptional repression, less is known about the biological consequences of differential DNA methylation outside gene promoter regions [21]. Increasing evidence, however, suggests that differential methylation in gene body regions may also play critical roles in gene regulation [20, 21]. Gene body methylation has been both positively and inversely associated with mRNA expression, and the direction of the effect may depend on the location of the aberrantly methylated CpG sites in the gene body [20, 21, 31]. The present study, therefore, supports a role for *CACNA1D* in PCa and provides evidence suggesting that overexpression of *CACNA1D* in *T2E*-positive tumors may result from hypomethylation of a CpG island in the gene body. These findings may have consequences for the treatment of PCa. In particular, there is some recent evidence suggesting that *CACNA1D* overexpression may induce prostate carcinogenesis and that these cancer-promoting effects may be counteracted by inhibition of the gene or the protein it encodes [7]. Further, a number of other recent studies provided evidence for a link between aberrant calcium-channel functioning and PCa [32–34].

The two other top-ranked hypomethylated CpGs in this study were in the gene body of *RAB40C* and the 5′ UTR of *GREM1*. While *RAB40C* methylation was not correlated with gene expression, *GREM1* showed higher mRNA transcript levels in fusion-positive PCa as compared to fusion-negative PCa. *GREM1* encodes a member of the bone morphogenic protein antagonist family [35]. *RAB40C* is a member of the *RAS* oncogene family, but the gene has not been well characterized [36].

The 19 hypermethylated CpGs in fusion-positive versus fusion-negative PCa included 15 CpGs in six genes: *PDE4D*, *SEPT9*, *NT5C*, *C3orf14*, *KLK10*, and *TRIB2*; and four intergenic CpGs including three CpGs near each other on chromosome 17. Six hypermethylated CpGs were in *PDE4D* (gene body or transcription start site). These CpGs were in or near the same CpG island, and their methylation levels were correlated. Phosphodiesterase 4D (PDE4D) may induce PCa cell proliferation [37], and one recent study showed that PDE4D inhibitors reduce prostate tumor growth in animal models [38]. Phosphodiesterases play important roles in cellular signaling [38].

*SEPT9* is a member of the septin family [39]. In the present study, *SEPT9* was associated with hypermethylation (promoter and gene body region) in fusion-positive PCa as compared to fusion-negative tumors, and gene expression was also higher in fusion-positive cases. While promoter hypermethylation is typically associated with transcriptional repression, a number of mechanisms via which gene body methylation changes may increase transcriptional activity have been suggested including blocking the initiation of intragenic promoters and affecting the activity of repetitive DNA elements within the transcriptional unit [31]. In a previous analysis, our group showed *SEPT9* hypermethylation in PCa compared to adjacent benign prostate tissue [40]. Furthermore, previous studies have identified hypermethylation of the *SEPT9* promoter region as a common event in a number of other cancers, and a diagnostic test that measures *SEPT9* methylation levels has been developed for colorectal cancer [41]. This study also showed promoter hypermethylation of *KLK10* and *TRIB2*, and *TRIB2* transcript levels were lower in *T2E*-positive PCa. *KLK10* is a member of the kallikrein family, which also includes *KLK3*, the gene that encodes prostate-specific antigen (PSA) [42]. A recent study showed that CpG methylation of *KLK10* was higher in prostate tumor compared to normal tissue and also reported an association between DNA methylation and clinicopathological parameters [43]. *TRIB2* plays a role in signal transduction pathways [44].

The gene *C3orf14* exhibited both promoter region hypermethylation and a corresponding strong decrease in mRNA expression. Although the function of this gene is unknown, a previous genome-wide analysis of glioblastoma versus normal brain tissue showed an inverse correlation between promoter region CpG methylation and mRNA expression of *C3orf14* [45].

Lastly, the gene *NTC5* had gene body hypermethylation and a decrease in mRNA expression. This gene encodes an enzyme that is critical for the physiological control of energy balance, metabolic regulation, and cell replication [46]. In summary, several of the top-ranked differentially methylated genes in the present study have molecular functions that suggest they may play a role in PCa. Further studies are needed to understand the specific mechanisms that underlie the link between differential DNA methylation and altered mRNA expression in these genes and prostate carcinogenesis.

Strengths of our study include the relatively large sample size, the epigenome-wide approach to identify differentially methylated CpG sites, and the ability to stratify patients by tumor *T2E* status as determined by FISH, which is considered the “gold standard” for measuring the gene fusion [47]. In addition, gene expression data from the same patients’ tumors were available to evaluate the potential biological effects of aberrant DNA methylation. We also used TCGA data to confirm our methylation findings. One potential limitation of this analysis is that *T2E* status was not directly measured using FISH in TCGA. We therefore used *ERG* mRNA expression to predict fusion status, and this indirect approach might have resulted in some misclassification. However, previous studies showed high concordance with *T2E* status as assessed by FISH and *ERG* mRNA expression [26–28]. Further, although we confirmed that our top results were similar in subgroups of European and African ancestry patients, the analysis of African-American men may have been underpowered due to small sample size.

## Conclusions

We report significant changes in the DNA methylome of *T2E*-positive versus *T2E*-negative prostate tumors. DNA methylation profiles were able to accurately distinguish between these major PCa subtypes. Results from our study were independently validated in TCGA. Several of the top-ranked differentially methylated genes in our study also showed mRNA expression changes, thereby providing evidence of an effect of aberrant DNA methylation on gene expression. These genes may play an important role in prostate carcinogenesis and highlight novel therapeutic targets that are specific for fusion-positive PCa. The findings from this study show that fusion-positive and fusion-negative PCa are epigenetically distinct, thereby providing further evidence that these unique molecular subtypes involve distinct alterations in disease pathways.

## Methods

### Prostate cancer patients

Data and tumor tissue samples were available from a cohort of patients who had radical prostatectomy as primary treatment for clinically localized PCa and who participated in one of two prior population-based studies [48, 49]. Baseline patient data were collected using an in-person interview. Information on clinicopathological parameters (e.g., Gleason score, disease stage, diagnostic prostate-specific antigen (PSA) level) was obtained from the Seattle-Puget Sound Surveillance, Epidemiology, and End Results (SEER) cancer registry. All patients signed informed consent, and procedures were approved by the Institutional Review Board of the Fred Hutchinson Cancer Research Center (Seattle, WA).

### Sample preparation

Formalin-fixed, paraffin-embedded (FFPE) blocks from radical prostatectomy specimens were used to make hematoxylin and eosin (H&E)-stained slides, which were reviewed by a PCa pathologist to confirm the presence and location of PCa within the blocks. Areas containing ≥75 % cancer cells had two 1-mm tumor tissue cores taken for DNA extraction, two for RNA extraction and two for tissue microarray (TMA) and immunohistochemistry analysis. In addition, for 20 patients (13 *T2E*-positive and 7 *T2E*-negative), adjacent non-tumor (histologically benign) prostate tissue cores were taken using the same procedure, and these samples were used for epigenome-wide DNA methylation profiling. Extraction of tumor DNA from the cores was completed using the RecoverAll Total Nucleic Acid Isolation Kit (Ambion/Applied Biosciences, Waltham, MA). The standard manufacturer’s protocol was followed, except that the elution step was performed twice to maximize DNA yield. Purified DNA was quantified (PicoGreen). RNA was isolated using the RNeasy® FFPE Kit (Qiagen Inc., Valencia, CA) and quantified using RiboGreen. DNA and RNA samples were stored at −80 °C and shipped to Illumina, Inc. (San Diego, CA) for completion of assays.

### DNA methylation arrays

Samples were bisulfite-converted using the EZ DNA Methylation Kit (Zymo Research, Irvine, CA) according to the manufacturer’s protocol. Controls on the array were used to track the bisulfite conversion efficiency. The Infinium HumanMethylation450 (HM450) BeadChip array (Illumina, Inc.) was used to measure epigenome-wide DNA methylation using beads with target-specific probes designed to interrogate individual CpGs (*n* >480,000) on bisulfite-converted genomic DNA. Duplicate samples for 16 patients were used, and these samples were randomly assigned to different plates. In addition, replicate tumor DNA samples from two patients were placed on every plate. All plates also contained Illumina controls and two negative controls. Laboratory personnel were blinded to patient characteristics (e.g., *T2E* status) as well as to the location of duplicate and replicate samples on plates. Samples were excluded if less than 95 % of the CpGs on the array for that sample were detected with a detection *P* value <0.05, which resulted in the exclusion of 33 samples (5.9 %). In total, 523 patients had available DNA methylation data. Correlations between blind duplicates ranged from 0.96 to 0.99 and were >0.99 for replicates across plates.

### Gene expression arrays

Expression profiling was done at Illumina using the Whole-Genome DASL^®^ (cDNA-mediated Annealing, Selection, Extension, and Ligation) HT Assay (Illumina, Inc.). Blind duplicate samples for six patients were randomly distributed across plates. Four samples failed, leaving 501 patients with available gene expression data. Transcript correlations between duplicated samples ranged from 0.96 to 0.99. In addition, replicate tumor RNA samples from two patients were included on every plate, and the transcript correlations across plates were 0.95 for each subject.

### Determination of *TMPRSS2*:*ERG* fusion status

Fluorescence in situ hybridization “break-apart” assays were used to determine *T2E*-fusion status [50]. A two-color fluorescence in situ hybridization technique was used, and the green fluorescein isothiocyanate signals were amplified with goat anti-fluorescein isothiocyanate Fluorescein/Oregon Green Antibody, Alexa Fluor 488 conjugate (Life Technologies, Waltham, MA) antibodies. Pictures were made with a Zeiss Axioplan 2 imaging system (Carl Zeiss AG) using Metafer (MetaSystems Inc., North Royalton, OH) imaging software. A 4′,6-diamidino-2-phenylindole prescan (×10 magnification) of the whole tumor tissue microarray slide was used to identify the core positions. Core identification numbers were assigned using a tumor tissue microarray tool implemented in Metafer. Each core was scanned at ×40 magnification, in a 6 × 9 grid of 54 fields. Each field was photographed in at least three different focus planes with filters for fluorescein isothiocyanate and cyanine 3. Referring layer and filter captures were then merged into one final three-colored image per field. Each core was evaluated by two separate individuals to determine whether the specimen was fusion-positive or fusion-negative. If there was disagreement, the specimen was reviewed until consensus was reached. Forty-eight (7.9 %) cases were excluded because cores could not be evaluated. Cores were considered positive if multiple cells contained the *T2E* rearrangement. For 38 (6.7 %) cases, *T2E* status had been determined using fluorescence in situ hybridization for a prior analysis [51], and these data were included. In total, 496 patients had both *T2E*-fusion status determined and DNA methylation data, and 467 patients had both *T2E* status determined and gene expression data.

### The Cancer Genome Atlas prostate cancer data

Data from The Cancer Genome Atlas (TCGA) were used to verify the most significant methylation results. HM450 data (level 3) were downloaded from the TCGA data portal (https://tcga-data.nci.nih.gov/tcga/). *T2E* status was not directly measured in TCGA, but previous studies have shown that *ERG* mRNA overexpression is an accurate predictor of positive fusion status [26–28]. We therefore analyzed TCGA PCa (exon) expression data (Illumina HiSeq; log_2_-normalized), which were downloaded from the UCSC (University of California, Santa Cruz) Cancer Browser (https://genome-cancer.ucsc.edu/). The mean *ERG* expression level across all samples was 2.12 (standard deviation = 1.48), and samples with an *ERG* expression level higher than this mean value were classified as tumors with *ERG* overexpression. Visual inspection of the data showed that this mean level is an appropriate cut-point to identify tumors with ERG overexpression. Of the 468 total samples available for DNA methylation analysis, 187 showed *ERG* overexpression (40 %).

The TCGA cohort is not population-based but includes patients from at least 30 centers around the world. High Gleason grade tumors are overrepresented in TCGA. The number of patients with Gleason score ≤6, 7 (3 + 4), 7 (4 + 3), and ≥8 were 47 (10 %), 139 (30 %), 98 (21 %), and 184 (39 %), respectively. The Gleason score was not different between tumors with versus without *ERG* overexpression (*P* value >0.05).

### Data processing and statistical data analysis

The Bioconductor *minfi* package was used to analyze the HM450 data. CpGs with an average detection *P* value >0.01 (*n* = 3715) and non-CpG probes (*n* = 2799) were excluded, and 478,998 CpGs were available for analysis. The data were normalized using subset-quantile within array normalization (SWAN) [52], and potential batch effects were removed using ComBat [53]. Methylation β-values were calculated, which represent the methylation level at each CpG locus: [intensity of the methylated allele/(intensity of the unmethylated allele + intensity of the methylated allele + 100)]. β-values range from 0 (unmethylated) to 1 (100 % methylated) and were used to identify the mean percentage methylation difference between fusion-positive and fusion-negative PCa. Global methylation levels were calculated by taking the average methylation level across CpGs per genetic and epigenetic location. Methylation M-values were also calculated by taking the logit transformation of the β-values.

Linear regression (Bioconductor *limma* package) with an empirical Bayes approach and using methylation M-values was conducted to assess whether CpGs were associated with *T2E*-fusion status. Models were adjusted for age at diagnosis (years; continuous), race (African-American, European-American), Gleason score (≤6, 7 [3 + 4], 7 [4 + 3], ≥8), and study (study I, study II). The same approach was used to analyze the gene expression data. Linear models, adjusted for the same variables, were also used to detect global DNA methylation differences. Statistical models used to analyze TCGA data were adjusted for age and Gleason score but not race because of missing data. False-discovery rate (FDR) Q-values were calculated to control the proportion of false positives, and a Q-value of less than 0.00001 was considered statistically significant. A chi-square test was used to test whether the frequencies of evaluated, hypermethylated, and hypomethylated CpG sites by genetic and epigenetic locations were different. In secondary analyses, associations of the top-ranked CpGs with *T2E* status were studied in subgroups based on race, Gleason score, and age at diagnosis. In addition, associations between the top-ranked CpGs and Gleason score (≥8 vs. ≤6) were studied in subgroups defined by fusion status.

Annotation data for the HM450 array were used. A gene promoter region was defined as follows: TSS1500, TSS200, 5′ UTR, and exon 1. Manhattan and volcano plots and heat maps were constructed to visualize the data. Principal component analysis (*prcomp*) and clustering (*heatmap.2* in Bioconductor *gplots* package) were also used to examine DNA methylation profiles. Methylation M-values were input for these analyses. Gene ontology analysis was conducted using hypergeometric testing and the Bioconductor *GOstats* package. All statistical analyses were conducted using the R programming language (http://cran.r-project.org/) and Bioconductor packages (http://bioconductor.org/).

## Additional file

Additional file 1:
**List of 3101 top-ranked differentially methylated CpG sites.**
